# Hypoxia-inducible factor 1α exerts dual roles in bladder cancer progression through TIMP3-mediated regulation of angiogenesis and invasion

**DOI:** 10.1038/s41598-026-39635-9

**Published:** 2026-02-12

**Authors:** Xiaoxian Wang, Jian Guo, Ruoran Zhang, Yang Dong, Lin Hao, Bo Chen, Hao Xu, Yuyang Ma, Kun Pang

**Affiliations:** 1 Department of Urology, Xuzhou Clinical College of Xuzhou Medical University, Southeast University Xuzhou Central Hospital, No.199, South Jiefang Road, Xuzhou, Jiangsu China; 2 Department of Urology, Zhongwu Hospital, No. 86, Xiamen Road, Suqian, Jiangsu China; 3 Graduate School, Jiangsu University, No. 301, Xuefu Road, Zhenjiang, Jiangsu China; 4 Department of Emergency, Southeast University Xuzhou Central Hospital, Xuzhou Clinical School of Xuzhou Medical College, No.199, South Jiefang Road, Xuzhou, Jiangsu China; 5 Department of Intensive Care Unit, Southeast University Xuzhou Central Hospital, Xuzhou Clinical School of Xuzhou Medical College, No.199, South Jiefang Road, Xuzhou, Jiangsu China

**Keywords:** Cancer, Cell biology, Oncology, Urology

## Abstract

Bladder cancer is one of the most prevalent malignancies of the urinary system, and its global incidence is rising, particularly among males. Hypoxia-inducible factor 1α (HIF-1α) plays a central role in enabling tumor cells to adapt to hypoxic conditions; however, its specific mechanisms of action in bladder cancer remain incompletely understood. This study aimed to investigate the effects of HIF-1α knockdown on bladder cancer cell proliferation, apoptosis, migration, invasion, and angiogenesis. The T24 and 5637 bladder cancer cell lines were employed. Specific si-HIF-1α plasmids were constructed and transfected into the cells. A series of functional assays were conducted, including MTT viability tests, flow cytometry for apoptosis analysis, Transwell migration and invasion assays, and in vitro angiogenesis experiments. Western blotting was performed to analyze the expression of tissue inhibitor of metalloproteinase 3 (TIMP3). Subsequent functional rescue experiments were carried out by overexpressing TIMP3. Additionally, clinical tissue samples were collected to assess the levels of HIF-1α and TIMP3. The results showed that under hypoxic conditions, knockdown of HIF-1α enhanced bladder cancer cell proliferation, migration, invasion, and angiogenesis while suppressing apoptosis. Furthermore, hypoxia was found to suppress TIMP3 expression in HIF-1α-knockdown cells. Notably, overexpression of TIMP3 reversed the alterations in bladder cancer cell behavior induced by HIF-1α knockdown. Analysis of clinical samples revealed that HIF-1α levels were significantly elevated in bladder cancer tissues. These findings uncover a complex regulatory role for HIF-1α in bladder cancer and identify TIMP3 as a critical mediator of its effects. Further studies are warranted to elucidate the transcriptional regulation of TIMP3 by HIF-1α and to validate its role in vivo, which may contribute to the advancement of targeted therapeutic strategies for bladder cancer.

**Supplementary Information:**

The online version contains supplementary material available at 10.1038/s41598-026-39635-9.

## Introduction

Bladder cancer, one of the most prevalent malignancies in the urinary system, exhibits a rising global incidence^1^, with a notably higher prevalence in males^2^. Global estimates indicate nearly one million new bladder cancer cases are diagnosed annually^2^, with the majority classified as urothelial carcinoma^3^. Current therapeutic approaches—including transurethral resection, Bacillus Calmette-Guérin (BCG) immunotherapy, and chemotherapy—significantly improve survival rates for patients with early-stage disease^4,5^. However, advanced cases face considerable challenges such as high rates of recurrence, metastasis, and the development of drug resistance^6^, resulting in a median overall survival of only a few months^7^. Therefore, elucidating the molecular mechanisms that drive bladder cancer progression and identifying novel therapeutic targets remain critical research priorities.

Hypoxia, a hallmark of the tumor microenvironment, plays a pivotal role in cancer progression^8^. Rapid tumor growth outpaces the often dysfunctional vascular supply, creating localized regions of oxygen deprivation. Under such conditions, tumor cells activate the hypoxia-inducible factor (HIF) signaling pathway to adapt and survive^9^. HIF-1α, a central mediator of this pathway, stabilizes under hypoxia and drives transcriptional programs that promote metabolic reprogramming^10^, angiogenesis, invasion, metastasis, and immune evasion^11,12^. For instance, HIF-1α upregulates vascular endothelial growth factor (VEGF) to stimulate angiogenesis^13^ and activates epithelial-mesenchymal transition (EMT) regulators such as Snail and Twist to enhance cell migratory capacity^14^.

In bladder cancer, HIF-1α exhibits context-dependent roles. While some studies link its overexpression to increased tumor aggressiveness^15^, others suggest it may exert paradoxical tumor-suppressive effects through specific signaling axes^16^. This functional duality implies the existence of a complex regulatory network that requires further exploration. Tissue inhibitor of metalloproteinase 3 (TIMP3), a key regulator of extracellular matrix homeostasis, inhibits matrix metalloproteinase (MMP) activity, thereby suppressing tumor invasion and angiogenesis^17^. Emerging evidence indicates that TIMP3 is dynamically regulated by hypoxia, and its loss correlates with increased metastatic potential in multiple cancers^18^. Whether HIF-1α modulates TIMP3 to influence bladder cancer progression remains unknown. Clarifying this interaction could bridge our understanding of hypoxia signaling with stromal remodeling mechanisms.

This study investigates the HIF-1α–TIMP3 axis in hypoxia-driven bladder cancer progression. Using HIF-1α knockdown models, we systematically assessed its effects on tumor cell proliferation, apoptosis, migration, invasion, and angiogenesis. Clinical tissue analyses further examined HIF-1α and TIMP3 expression patterns, offering insights into their prognostic relevance and therapeutic potential.

## Materials and methods

### Cell lines and culture conditions

The study utilized two human bladder cancer cell lines, T24 and 5637, obtained from the Cell Bank of the Chinese Academy of Sciences. Cells were maintained in RPMI-1640 medium supplemented with 10% fetal bovine serum (FBS), 1% penicillin-streptomycin, and 1% L-glutamine. All cultures were incubated at 37 °C in a humidified atmosphere containing 5% CO₂. To mimic tumor microenvironment conditions, cells were exposed to 1% O₂ in a dedicated hypoxic chamber for hypoxia experiments. Normoxic control cells were cultured under standard atmospheric oxygen levels (21% O₂). Cells were passaged every 2–3 days, and all experiments were conducted when cells reached approximately 80% confluency.

### Plasmid construction and transfection

To investigate the role of HIF-1α in bladder cancer cells, a specific si-HIF-1α plasmid was designed for targeted knockdown. Synthetic siRNA sequences were integrated into the plasmid backbone using standard molecular cloning techniques. Cells were seeded in 6-well plates and transfected at 60–80% confluency using Lipofectamine^®^ 3000 reagent (Thermo Fisher Scientific, USA) according to the manufacturer’s protocol. Briefly, siRNA-Lipofectamine complexes were prepared and added to each well, gently mixed, and incubated with the cells. Post-transfection, cells were maintained at 37 °C with 5% CO₂ for 24 h. A GFP-tagged plasmid was used as a transfection control, and efficiency was confirmed via fluorescence microscopy. HIF-1α expression levels were quantified via quantitative PCR (qPCR) 48 h post-transfection to validate knockdown efficiency. The siRNA sequence demonstrating the highest silencing efficacy was selected for all subsequent experiments.

### Cell-based functional assays

Multiple assays were performed to evaluate the effects of HIF-1α knockdown on bladder cancer cell proliferation, apoptosis, migration, invasion, and angiogenic potential. Experimental groups included HIF-1α knockdown cells cultured under both normoxic and hypoxic conditions.

#### MTT assay for cell proliferation

Cell proliferation was assessed using the MTT assay. Transfected cells were seeded in 96-well plates at a density of 3,000 cells per well and incubated for 24 h. Following culture under normoxic or hypoxic conditions for 24, 48, or 72 h, cells were treated with 20 µL of MTT solution (5 mg/mL in PBS) and incubated for an additional 4 h. The resulting formazan crystals were dissolved with 150 µL of dimethyl sulfoxide (DMSO), and the absorbance at 570 nm was measured using a BioTek microplate reader (USA). Absorbance values across the different time points were compared to evaluate hypoxia-induced changes in proliferation following HIF-1α knockdown.

#### Apoptosis analysis by flow cytometry

Apoptosis was assessed using Annexin V-FITC/PI double staining. Cells cultured under normoxic or hypoxic conditions for 48 h were harvested, washed with PBS, and resuspended in 1× binding buffer. Staining was performed following the protocol of the Annexin V-FITC/PI apoptosis detection kit. Flow cytometry analysis was conducted using a BD FACS Calibur instrument (USA) to quantify the percentages of early-stage (Annexin V-FITC-positive) and late-stage (Annexin V-FITC/PI-double-positive) apoptotic cells.

#### Transwell migration and invasion assays

Transwell chambers with 8 μm pores were used for migration and invasion assays. For the migration assay, 1 × 10⁵ transfected cells in serum-free medium were seeded into the upper chamber, while medium containing 10% FBS was placed in the lower chamber as a chemoattractant. After 24 h of incubation, cells that had migrated to the lower surface of the membrane were fixed, stained with 0.1% crystal violet, and counted under a microscope. For the invasion assay, Matrigel-coated inserts were employed, and cells were incubated for 48 h before staining and quantification. Results were analyzed to assess hypoxia-mediated changes in migratory and invasive capacities under conditions of HIF-1α knockdown.

#### Angiogenesis assay

Angiogenic potential was evaluated using a human umbilical vein endothelial cell (HUVEC) tube formation assay. Conditioned medium was collected from transfected bladder cancer cells after 48 h of culture, centrifuged to remove debris, and then added to HUVEC cultures. HUVECs (1 × 10⁴ cells per well) were seeded in 24-well plates and incubated for 24 h. The resulting tubular structures were imaged under a microscope, and angiogenesis was quantified by measuring the number of tubes and their total length. This assay assessed the hypoxia-driven angiogenic effects mediated by HIF-1α-knockdown bladder cancer cells.

### Western blot analysis of TIMP3 expression

Western blotting was performed to assess TIMP3 expression in HIF-1α-knockdown bladder cancer cells under normoxic and hypoxic conditions. Cells were cultured for 48 h, lysed, and the supernatants were collected after centrifugation (12,000 rpm, 4 °C, 10 min). Protein concentrations were quantified using the bicinchoninic acid (BCA) method. Equal amounts of protein (30 µg) were loaded onto 12% SDS-PAGE gels for separation. Subsequently, proteins were transferred to polyvinylidene difluoride (PVDF) membranes. The membranes were blocked with 5% skim milk in PBS for 1 h at room temperature and then incubated overnight at 4 °C with primary antibodies against TIMP3 and β-actin (1:1000 dilution). After washing with TBST, membranes were incubated with horseradish peroxidase (HRP)-conjugated secondary antibodies (1:5000 dilution) for 1 h. Protein bands were visualized using enhanced chemiluminescence (ECL) substrate on a Bio-Rad imaging system (USA). Band densities for TIMP3 were quantified using ImageJ software and normalized to β-actin to evaluate the effects of HIF-1α knockdown.

### Functional rescue experiment

To confirm the specific role of TIMP3 in mediating the effects observed in HIF-1α-knockdown cells, functional rescue experiments involving TIMP3 overexpression under hypoxic conditions were conducted. The experimental groups were as follows: si-HIF-1α (normoxia), si-HIF-1α + pcDNA3-NC (empty vector control, hypoxia), and si-HIF-1α + pcDNA3-TIMP3 (recombinant TIMP3 overexpression plasmid, hypoxia).

#### MTT assay for proliferation rescue

MTT assays were performed to evaluate cell proliferation across the different experimental groups. Cells (3,000 per well) were seeded in 96-well plates and cultured under normoxia or hypoxia for 24 to 72 h. After adding MTT solution (5 mg/mL in PBS), cells were incubated for 4 h, followed by solubilization with DMSO. Absorbance at 570 nm was measured to assess the ability of TIMP3 overexpression to rescue the effects of HIF-1α knockdown on proliferation.

#### Apoptosis analysis by flow cytometry

Apoptosis was analyzed using Annexin V-FITC/PI double staining. Cells were harvested 48 h post-transfection, washed with PBS, resuspended in 1× binding buffer, and stained according to the kit protocol. Flow cytometry was used to quantify the percentages of early apoptotic (Annexin V-FITC-positive) and late apoptotic (Annexin V-FITC/PI-double-positive) cells.

#### Transwell migration and invasion rescue assays

Transwell chambers were used to assess the ability of TIMP3 overexpression to rescue changes in migration and invasion. For the migration assay, cells in serum-free medium were seeded into uncoated inserts, with medium containing 10% FBS as a chemoattractant in the lower chamber. After 24 h, migrated cells were fixed and stained with 0.1% crystal violet. For the invasion assay, Matrigel-coated inserts were used, and cells were incubated for 48 h before fixation, staining, and quantification. Cells were counted under a microscope to evaluate TIMP3’s role in reversing the effects of HIF-1α knockdown on migration and invasion.

### Clinical tissue sample analysis

Bladder tumor tissues and matched adjacent normal bladder tissues were collected and analyzed to evaluate the clinical relevance of HIF-1α and TIMP3 expression in bladder cancer. All samples were collected with written informed consent from patients and the study protocol was approved by the Ethics Committee of Xuzhou Central Hospital. All methods were performed in accordance with the relevant guidelines and regulation. The procedures for sample collection and processing are detailed below.

#### Sample collection and preservation

 Tumor tissues and adjacent normal bladder tissues were obtained from patients undergoing radical cystectomy for bladder cancer. Samples were collected under sterile conditions to ensure quality and representativeness. Tissues were immediately snap-frozen in liquid nitrogen and stored at -80 °C until RNA or protein extraction to prevent degradation.

#### RNA extraction and qRT-PCR

Tissue samples were homogenized, and total RNA was extracted using TRIzol reagent (Invitrogen, USA). RNA concentration and purity were measured with a NanoDrop spectrophotometer (Thermo Fisher Scientific, USA). Reverse transcription was performed using a commercial kit (Thermo Fisher Scientific) to generate complementary DNA (cDNA). The mRNA levels of HIF-1α and TIMP3 were quantified via quantitative real-time PCR (qRT-PCR) using gene-specific primers. Relative gene expression between tumor and normal tissues was calculated using the 2 − ΔΔCt method. 

#### Protein extraction and western blot

Proteins were extracted from homogenized tissue samples using RIPA lysis buffer. Protein concentrations were determined via the BCA assay. Equal amounts of protein were separated by SDS-PAGE, transferred to PVDF membranes, and blocked with 5% skim milk. Membranes were probed with primary antibodies against HIF-1α and TIMP3 overnight, followed by incubation with HRP-conjugated secondary antibodies. Protein bands were visualized using ECL substrate on a Bio-Rad imaging system (USA). Band intensities were quantified using ImageJ software to compare protein expression levels.

### Statistical analysis

Data were analyzed using SPSS software. Differences between experimental groups and control groups were assessed using the Student’s t-test. Results are expressed as mean ± standard deviation (SD). A P-value of less than 0.05 was considered statistically significant. All experiments included three independent replicates to ensure reliability and reproducibility.

## Results

### HIF-1α is overexpressed in bladder cancer tissues

**Fig. 1:**
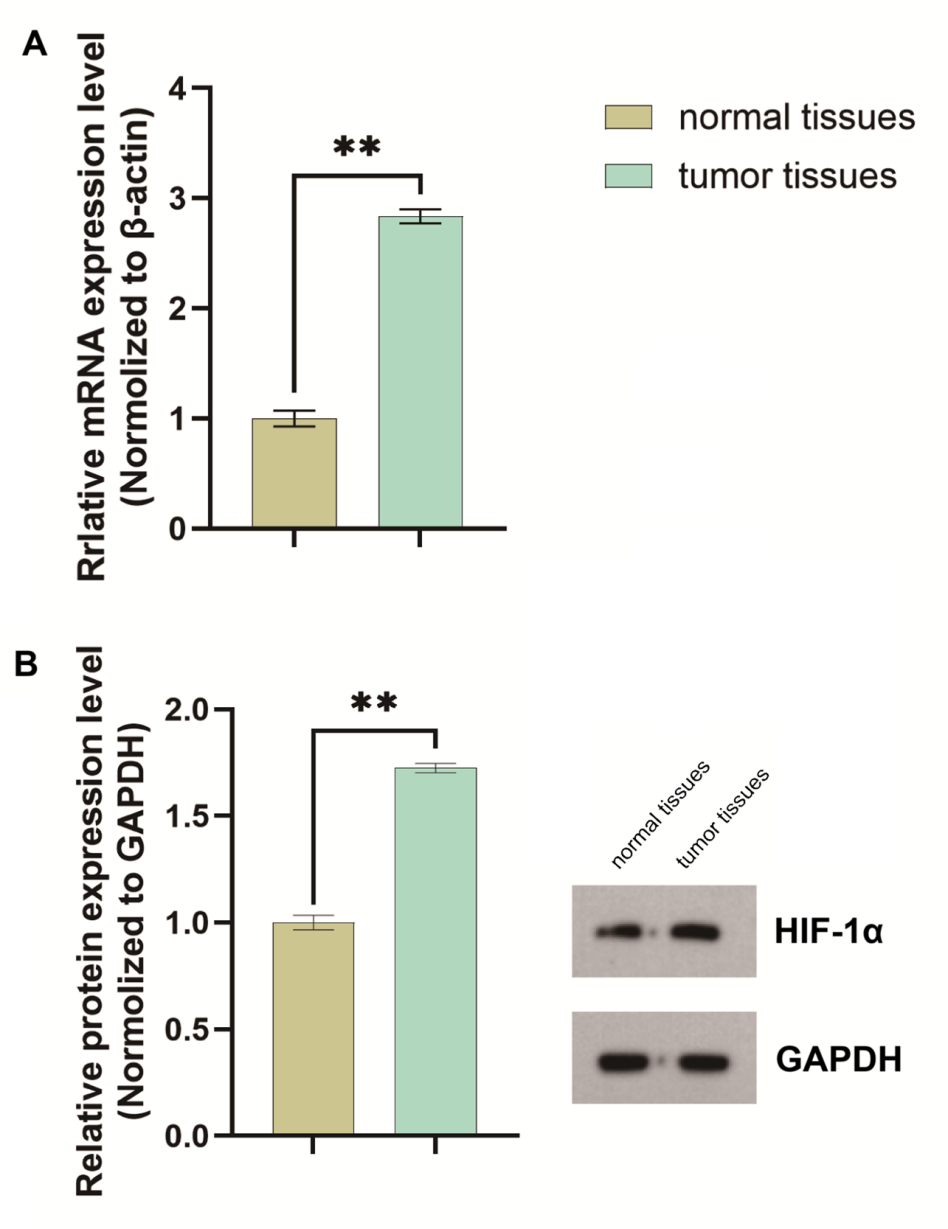
HIF-1α is overexpressed in bladder cancer cells. (**A**) qPCR analysis indicated that the HIF − 1α gene expression in bladder cancer tissues was 2.83 - fold higher than that in adjacent normal tissues(*P* < 0.05**). (**B**) Western blot analysis showed significantly higher HIF-1α protein levels in bladder cancer tissues compared to adjacent normal tissues (HIF-1α/GAPDH ratio: 0.95 vs. 0.55) (*P* < 0.05**). These experiments were repeated three times.

qPCR analysis revealed a 2.83-fold increase in HIF-1α gene expression in tumor tissues compared to adjacent normal tissues. Western blot results further demonstrated elevated HIF-1α protein levels in bladder cancer tissues. The HIF-1α/GAPDH ratio was 0.951315 in tumor tissues, which was significantly higher than the ratio in adjacent normal tissues (0.551904) (Fig. 1).

### HIF-1α was successfully knocked down by si-HIF-1α

**Fig. 2:**
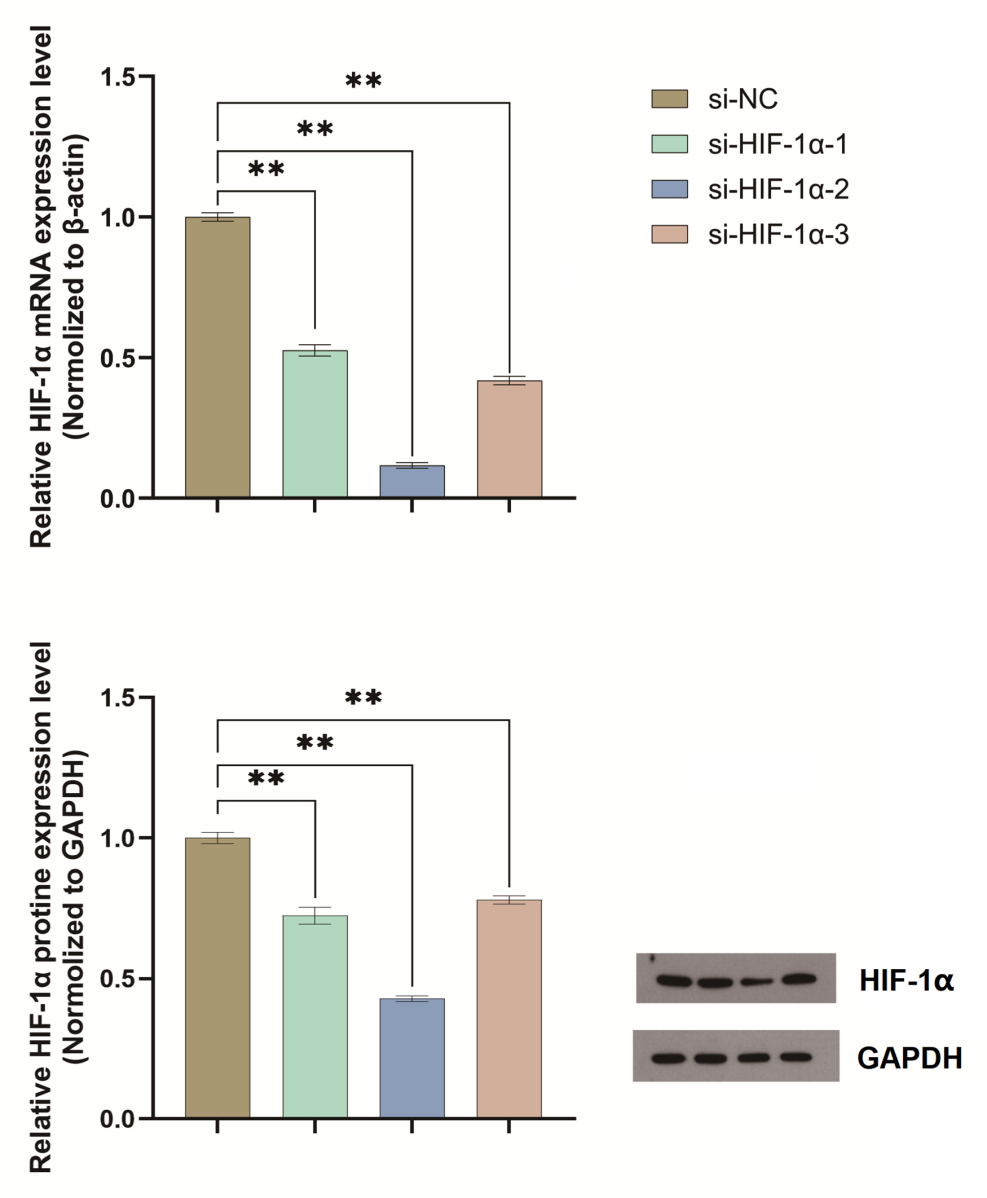
qPCR Validation of si-HIF-1α Knockdown efficiency. qPCR analysis and Western blot revealed that si-HIF-1α-2 significantly reduced HIF-1α expression compared to Si-NC, and was selected for further experiments(*P* < 0.05**). These experiments were repeated three times.

qPCR analysis confirmed efficient knockdown of HIF-1α using the constructed si-HIF-1α plasmids. Among the tested constructs, si-HIF-1α-2 exhibited the highest silencing efficacy. Western blot results provided quantitative support: In si-NC control cells, the grayscale values were 38,414 for GAPDH and 49,381 for HIF-1α, yielding a relative HIF-1α expression level (HIF-1α/GAPDH) of 1.285. In si-HIF-1α-1 cells, the values were 38,505 (GAPDH) and 35,814 (HIF-1α), with a relative expression of 0.930 (representing a 0.724-fold change compared to si-NC). In si-HIF-1α-2 cells, values were 37,849 (GAPDH) and 20,792 (HIF-1α), corresponding to a relative expression of 0.549 (a 0.427-fold change compared to si-NC). In si-HIF-1α-3 cells, values were 38,196 (GAPDH) and 38,255 (HIF-1α), resulting in a relative expression of 1.002 (a 0.780-fold change). Based on these results, si-HIF-1α-2 was selected for all subsequent functional assays (Fig. 2).

### HIF-1α knockdown regulates hypoxia-induced cell proliferation, apoptosis, migration, invasion, and angiogenesis

A series of functional assays evaluated the effects of HIF-1α knockdown on bladder cancer cells under hypoxia. The assays for proliferation, apoptosis, migration, invasion, and angiogenesis collectively revealed that silencing HIF-1α significantly enhanced proliferation, migration, invasion, and angiogenesis while suppressing apoptosis under hypoxic conditions.

#### Hypoxia enhances bladder cancer cell proliferation under HIF-1α knockdown

**Fig. 3:**
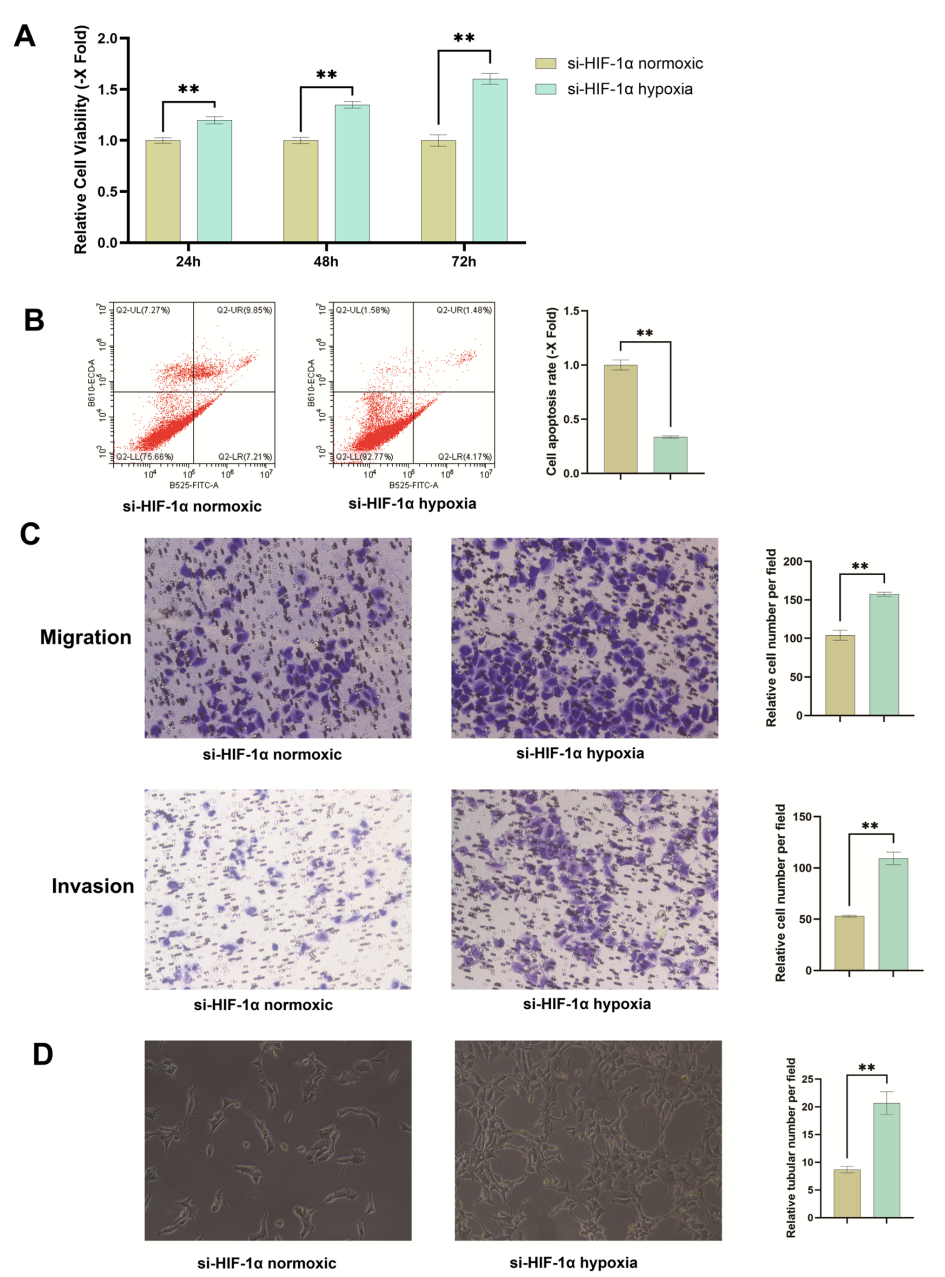
HIF-1α knockdown regulates hypoxia-induced cell proliferation, apoptosis, migration, invasion and angiogenesis. (**A**) MTT assay showed reduced cell viability under hypoxia in si-HIF-1α-transfected cells compared to normoxic conditions at 48 h and 72 h(*P* < 0.05**). (**B**) Total apoptosis decreased from 17.06% (normoxia) to 5.65% (hypoxia) in si-HIF-1α-transfected cells (fold change = 0.331), with early apoptosis (7.21% vs. 4.17%) and late apoptosis (9.85% vs. 1.48%) reduced(*P* < 0.05**). (**C**) shows hypoxia increased migration and invasion 1.51-fold and 2.06 -fold vs. normoxia in si-HIF-1α-transfected cells(*P* < 0.05**). (**D**) Hypoxia increased tubular network formation (vessel number and length) in HUVEC cells co-cultured with HIF-1α-silenced bladder cancer cells compared to normoxia(*P* < 0.05**). These experiments were repeated three times.

MTT assays evaluated proliferation across the experimental groups. HIF-1α-knockdown cells cultured under hypoxic conditions showed higher absorbance readings at 24, 48, and 72 h compared to normoxic controls, with the relative absorbance increasing over time. These results indicate that hypoxia promotes bladder cancer cell proliferation following HIF-1α knockdown, exhibiting a time-dependent enhancement (Fig. 3A).

#### HIF-1α knockdown suppresses apoptosis under hypoxia

Under conditions of HIF-1α knockdown, hypoxia reduced the rate of early apoptosis from 7.21% to 4.17% and late apoptosis from 9.85% to 1.48%. This resulted in a total apoptosis rate of 5.65% under hypoxia, compared to 17.06% under normoxia (fold change = 0.331). These data indicate that hypoxia suppresses total apoptosis by approximately 66.9% relative to normoxic conditions in HIF-1α-silenced cells (Fig. 3B).

#### HIF-1α knockdown enhances cell migration and invasion under hypoxia

Hypoxia increased Transwell migration by 1.51-fold and Matrigel invasion by 2.06-fold in HIF-1α-knockdown cells compared to normoxic controls. These findings demonstrate that hypoxia significantly promotes both the migratory and invasive capacities of bladder cancer cells under conditions of HIF-1α silencing (Fig. 3C).

#### HIF-1α knockdown enhances angiogenesis under hypoxia

Microscopic analysis revealed that HUVEC cells, when exposed to conditioned medium from hypoxic HIF-1α-knockdown bladder cancer cells, formed more extensive tubular networks. Significant increases in both vessel number and total vessel length were observed compared to HUVECs treated with conditioned medium from normoxic controls. These findings demonstrate that HIF-1α silencing under hypoxic conditions potentiates the angiogenic potential of bladder cancer cells (Fig. 3D).

### Hypoxia suppresses TIMP3 protein expression in HIF-1α-knockdown cells

**Fig. 4:**
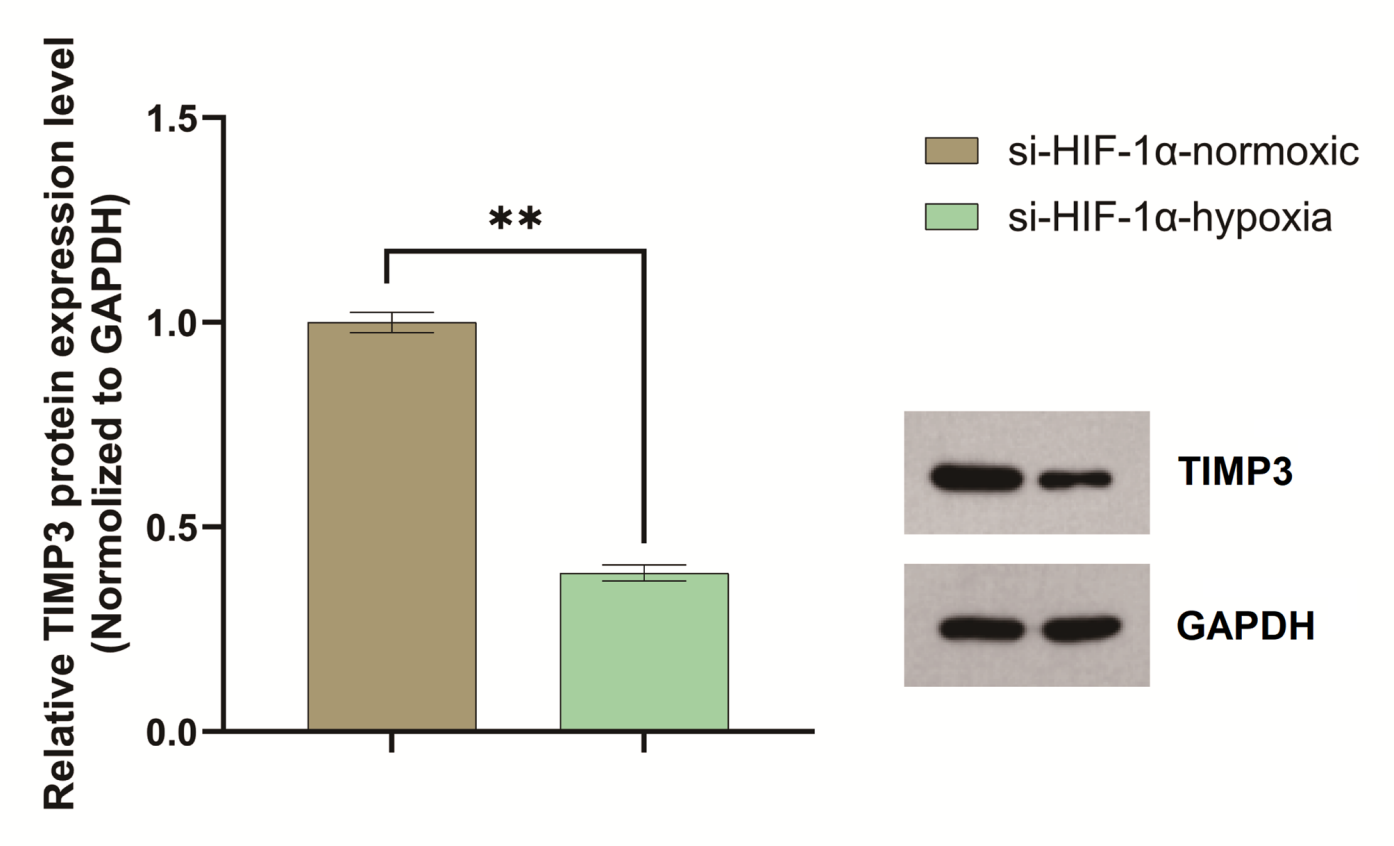
Hypoxia downregulates TIMP3 expression under HIF-1α knockdown. TIMP3 protein levels (normalized to GAPDH) decreased by 27.6% (fold change = 0.72) in HIF-1α knockdowd cells under hypoxia compared to normoxia(*P* < 0.05**). These experiments were repeated three times.

Under normoxic conditions, the normalized TIMP3 protein expression (TIMP3/GAPDH) in HIF-1α-knockdown cells was 1.09 (GAPDH grayscale: 57,228; TIMP3 grayscale: 62,408). Exposure to hypoxia reduced the normalized TIMP3 expression to 0.42 (GAPDH grayscale: 57,715; TIMP3 grayscale: 24,421), representing a 61.2% decrease (fold change = 0.38). These results demonstrate that hypoxia suppresses TIMP3 expression in HIF-1α-silenced cells (Fig. 4).

### TIMP3 overexpression reverses hypoxia-driven phenotypes in HIF-1α knockdown cells

**Fig. 5:**
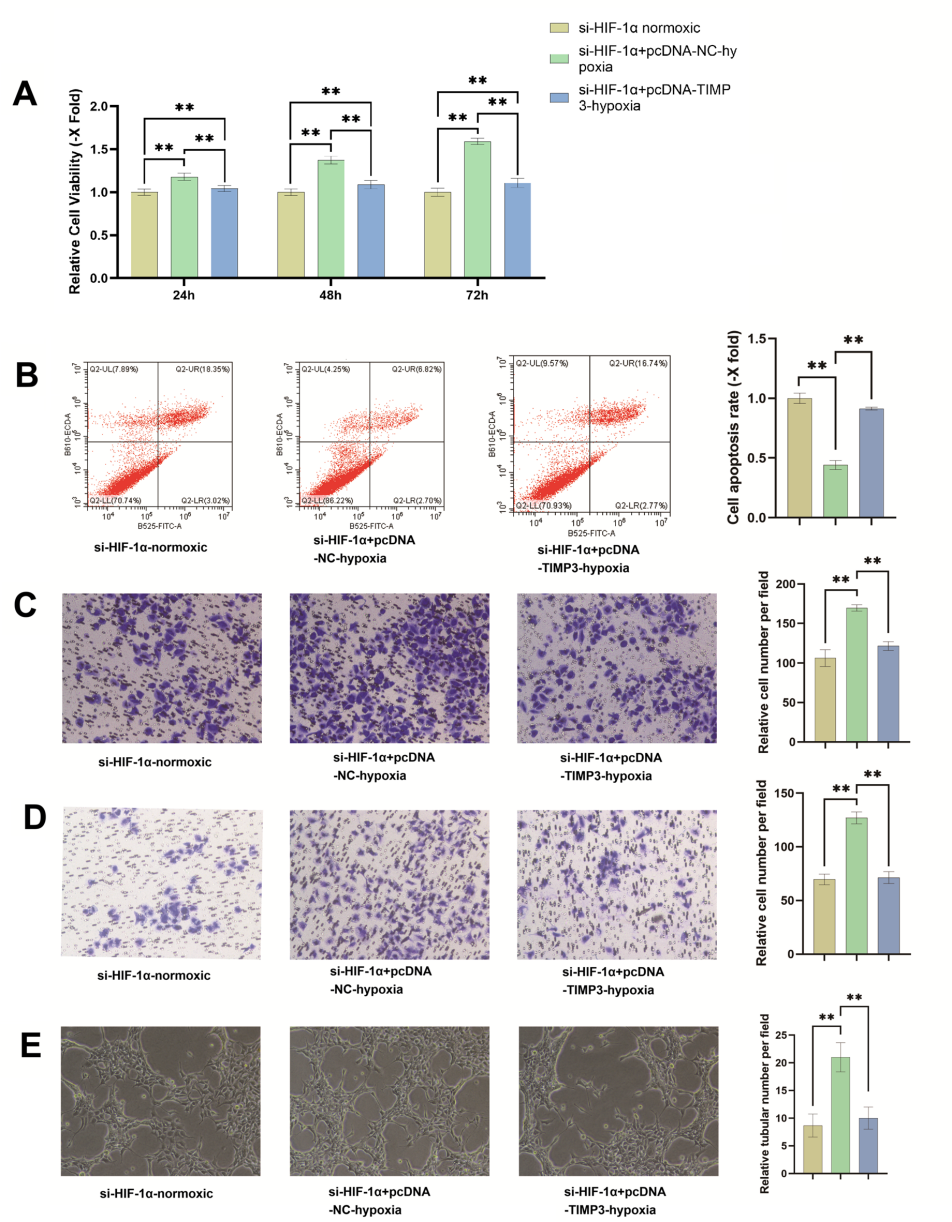
TIMP3 overexpression reversed hypoxia-driven proliferation, migration, invasion and angiogenesis in HIF-1α knockdown cells. (**A**) MTT assays revealed hypoxia-enhanced cell viability in si-HIF-1α + pcDNA-NC cells versus normoxic controls. TIMP3 overexpression (si-HIF-1α + pcDNA-TIMP3-hypoxia) partially reversed hypoxia-induced proliferation, showing intermediate viability(*P* < 0.05**). (**B**) TIMP3 overexpression increased total apoptosis from 9.52% to 19.51% in hypoxic si-HIF-1α cells (fold change = 0.913 vs. 0.445), nearing normoxia levels(*P* < 0.05**). (**C**) Migration of si-HIF-1α + pcDNA3-TIMP3-hypoxia cells decreased to 121.67 (vs. 169.67 in si-HIF-1α + pcDNA3-NC-hypoxia; fold change = 1.14 vs. 1.59), approaching normoxic levels(*P* < 0.05**). (**D**) Invasion of si-HIF-1α + pcDNA3-TIMP3-hypoxia cells was reduced to 71.33 (vs. 127 in si-HIF-1α + pcDNA3-NC-hypoxia; fold change = 1.02 vs. 1.82), comparable to normoxic controls(*P* < 0.05**). (**E**) TIMP3 overexpression significantly reduced tubular network formation (vessel number and length) in HUVECs co-cultured with HIF-1α-silenced bladder cancer cells under hypoxic conditions (*P* < 0.05**). These experiments were repeated three times.

Functional rescue experiments validated that TIMP3 overexpression could counteract the effects of HIF-1α knockdown under hypoxia. MTT assays showed lower absorbance values for cell proliferation in the si-HIF-1α normoxia group compared to the si-HIF-1α + pcDNA3-NC hypoxia group, confirming that hypoxia promotes proliferation. The si-HIF-1α + pcDNA3-TIMP3 hypoxia group exhibited intermediate absorbance values, which gradually increased over time but remained reduced relative to the si-HIF-1α + pcDNA3-NC hypoxia group. This suggests that TIMP3 overexpression partially counteracted the hypoxia-induced proliferation potentiated by HIF-1α knockdown (Fig. 5A). Flow cytometry revealed a significant increase in the total apoptosis rate in the si-HIF-1α + pcDNA3-TIMP3 hypoxia group (19.51%) compared to the si-HIF-1α + pcDNA3-NC hypoxia group (9.52%). The fold change (relative to normoxia) rose from 0.445 to 0.913, approaching the level observed under normoxia with HIF-1α knockdown. These results indicate that TIMP3 overexpression partially reversed the hypoxia-mediated suppression of apoptosis following HIF-1α knockdown (Fig. 5B). Migration and invasion assays demonstrated reduced mean migration values in the si-HIF-1α + pcDNA3-TIMP3 hypoxia group (121.67 vs. 169.67 in the si-HIF-1α + pcDNA3-NC hypoxia group; fold change relative to normoxia: 1.14 vs. 1.59). Similarly, invasion values decreased (71.33 vs. 127; fold change: 1.02 vs. 1.82). Both metrics approached the levels seen in the si-HIF-1α normoxia group, indicating that TIMP3 overexpression effectively reversed the hypoxia-enhanced migration and invasion caused by HIF-1α knockdown (Fig. 5C and D). Microscopic analysis of the tube formation assay revealed that HUVECs in the si-HIF-1α + pcDNA3-TIMP3 hypoxia group formed tubular structures with significantly reduced number and length compared to the si-HIF-1α + pcDNA3-NC hypoxia group. These parameters approached levels observed in the si-HIF-1α normoxia group. These findings indicate that TIMP3 overexpression effectively reversed, to a significant extent, the enhanced pro-angiogenic effects induced by HIF-1α knockdown under hypoxic conditions (Fig. 5E). Collectively, restoring TIMP3 expression partially or fully reversed the effects of HIF-1α knockdown on bladder cancer cell proliferation, apoptosis, migration, and invasion under hypoxia.

## Discussion

HIF-1α, a central regulator of the hypoxic tumor microenvironment, exhibits functional heterogeneity across different cancer types. This study systematically investigates its distinct role in bladder cancer under hypoxic conditions through targeted HIF-1α knockdown. Our results demonstrate that HIF-1α knockdown under hypoxia significantly increases bladder cancer cell proliferation, migration, invasion, and angiogenesis while inhibiting apoptosis. This finding contrasts with prior studies that primarily identify HIF-1α as a pro-tumorigenic factor, suggesting a context-dependent dual role in bladder cancer. While HIF-1α is known to promote tumor progression via classical pathways such as VEGF upregulation and EMT activation, its knockdown here paradoxically enhances malignant phenotypes. This implies that HIF-1α may also possess regulatory roles in suppressing alternative pro-tumor signals to maintain cellular homeostasis. For instance, the loss of HIF-1α may relieve its suppression of pro-apoptotic factors or inadvertently activate compensatory proliferation pathways (e.g., PI3K/AKT or MAPK)^19^, thereby accelerating tumor progression. This paradoxical phenomenon underscores the complexity of tumor signaling networks and necessitates microenvironment-specific analyses to fully elucidate HIF-1α’s functional dynamics.

A key finding of this study is that TIMP3 may serve as a critical downstream effector for the tumor-suppressive aspect of HIF-1α function. Under HIF-1α knockdown conditions, hypoxia markedly suppresses TIMP3 expression, whereas restoring TIMP3 reverses the pro-malignant effects induced by HIF-1α depletion. As a natural inhibitor of MMPs, reduced TIMP3 expression likely exacerbates extracellular matrix degradation, thereby facilitating tumor invasion and angiogenesis^17^. Our further analysis supports the notion that HIF-1α likely regulates TIMP3 expression through direct or indirect mechanisms, positioning TIMP3 as a functional link between the hypoxic microenvironment and matrix remodeling. However, the precise transcriptional regulatory mechanisms—such as potential direct binding of HIF-1α to hypoxia-response elements (HREs) in the TIMP3 promoter or its cooperation with other transcription factors (e.g., NF-κB)—require further dedicated investigation.

Clinical tissue analysis confirms elevated HIF-1α expression in bladder cancer tissues, which is consistent with a pro-tumorigenic role observed in part of our cellular experiments. While this aligns with prior reports of HIF-1α overexpression in various tumors^20^, the current study advances this understanding by linking its paradoxical context-dependent role to the diversity of its regulatory targets. These findings propose a refined therapeutic strategy: inhibition of HIF-1α alone may inadvertently disrupt its regulation of factors like TIMP3, potentially yielding limited or adverse effects. In contrast, a combination approach that suppresses HIF-1α while concurrently targeting downstream pro-tumor pathways (e.g., VEGF) and aiming to restore TIMP3 function could offer enhanced treatment precision and efficacy.

While this study advances the understanding of HIF-1α’s dual roles, certain limitations warrant consideration. Regarding mechanistic depth, although we have identified the effects of HIF-1α knockdown on multiple biological behaviors and its association with TIMP3 regulation, it remains unclear whether HIF-1α directly transcriptionally regulates TIMP3 and what other molecules are under its control in this context. In terms of preclinical relevance, the current findings primarily rely on in vitro cell experiments and clinical tissue sample analysis, lacking in vivo validation. Future work should incorporate studies in animal models to confirm the pro-tumorigenic effects of HIF-1α loss in a living organism. Such research will be crucial for developing more precise and effective bladder cancer treatment strategies.

In summary, this study uncovers a novel mechanism by which HIF-1α influences bladder cancer progression through the regulation of TIMP3, laying important theoretical groundwork for future therapies targeting the hypoxic tumor microenvironment. Future research should prioritize elucidating the detailed HIF-1α-TIMP3 transcriptional network and exploring its potential synergy with existing therapies, such as immune checkpoint inhibitors. This may open new avenues for innovative treatment strategies for bladder cancer based on a more comprehensive understanding of the complex regulatory network centered on HIF-1α.

## Conclusion

This study reveals a paradoxical role for HIF-1α in bladder cancer progression under hypoxic conditions. Knockdown of HIF-1α unexpectedly enhances tumor malignancy by promoting proliferation, migration, invasion, and angiogenesis while suppressing apoptosis. This aggressive phenotype is mediated, at least in part, through hypoxia-induced downregulation of TIMP3. Functional rescue experiments demonstrate that restoring TIMP3 expression reverses these aggressive behaviors, identifying TIMP3 as a critical downstream effector of HIF-1α signaling in this context. Clinically, elevated HIF-1α levels in bladder cancer tissues correlate with tumor progression, reinforcing its pathological relevance. These findings challenge the conventional purely oncogenic view of HIF-1α and highlight its context-dependent duality within hypoxic microenvironments. Future studies should delineate the precise transcriptional interplay between HIF-1α and TIMP3 and validate these mechanisms in vivo. Such work is essential for paving the way towards rational combination therapies that target both hypoxia signaling and matrix remodeling pathways in bladder cancer.

## Supplementary Information

Below is the link to the electronic supplementary material.

Supplementary Material 1

Supplementary Material 2

## Data Availability

The datasets generated and analyzed during the current study are not publicly available due to patient privacy considerations but are available from the corresponding author upon reasonable request. No sequencing, proteomics, or other high-throughput datasets were generated in this study.
