# Supplementary material for: Hypoxia-inducible factor 1α exerts dual roles in bladder cancer progression through TIMP3-mediated regulation of angiogenesis and invasion
Source: Sci Rep. 2026 Feb 12;16:8425. doi: 10.1038/s41598-026-39635-9 (PMC12972112; doi:10.1038/s41598-026-39635-9)
Supplement: Supplementary file 1 — Supplementary Material 1 [file 41598_2026_39635_MOESM1_ESM.pdf]

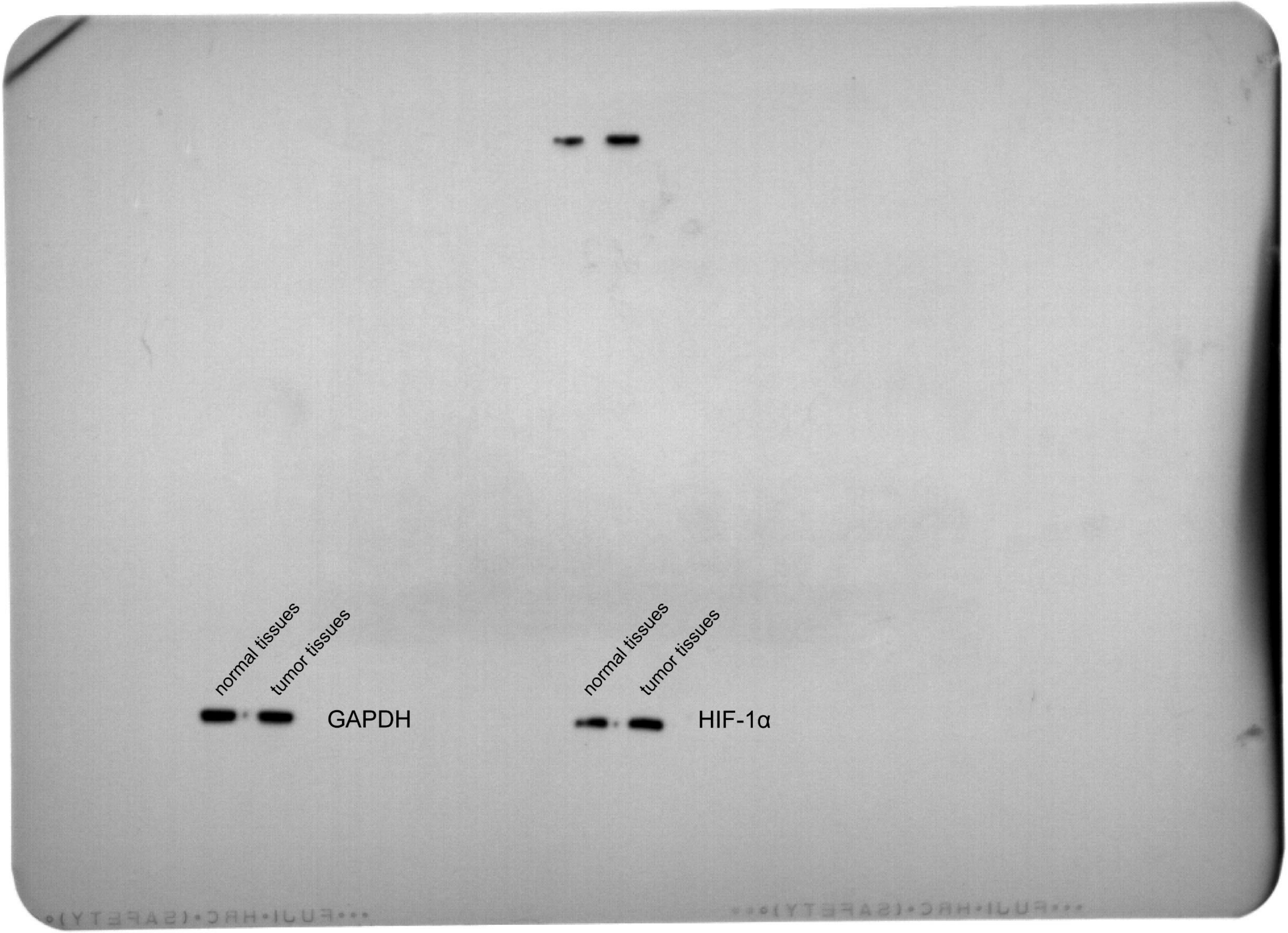
normal tissues
tumor tissues
GAPDH
normal tissues
tumor tissues
HIF-1α
