# Supplementary material for: Hypoxia-inducible factor 1α exerts dual roles in bladder cancer progression through TIMP3-mediated regulation of angiogenesis and invasion
Source: Sci Rep. 2026 Feb 12;16:8425. doi: 10.1038/s41598-026-39635-9 (PMC12972112; doi:10.1038/s41598-026-39635-9)
Supplement: Supplementary file 2 — Supplementary Material 2 [file 41598_2026_39635_MOESM2_ESM.pdf]

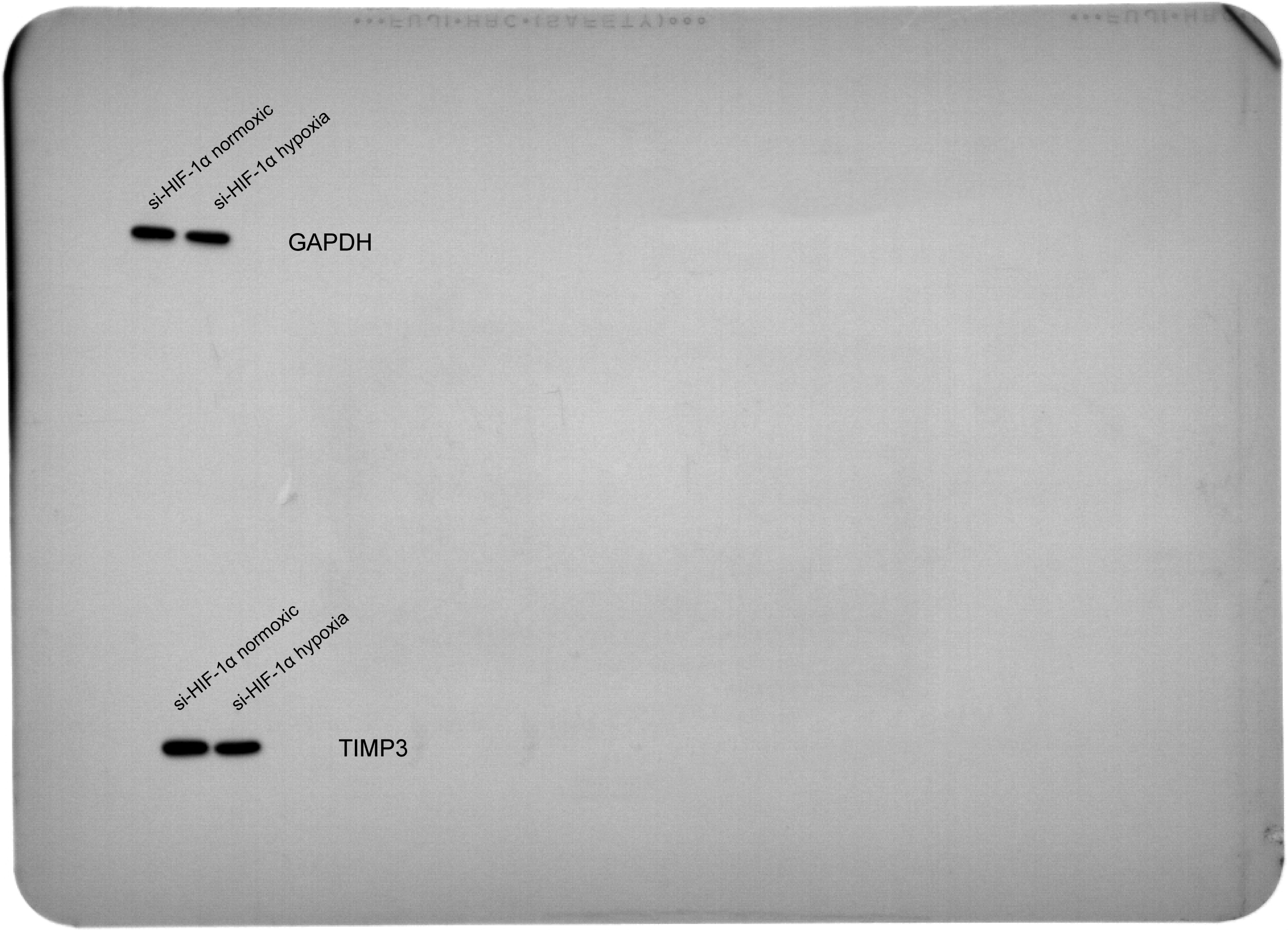
si-HIF-1α normoxic
si-HIF-1α hypoxia
GAPDH
si-HIF-1α normoxic
si-HIF-1α hypoxia
TIMP3

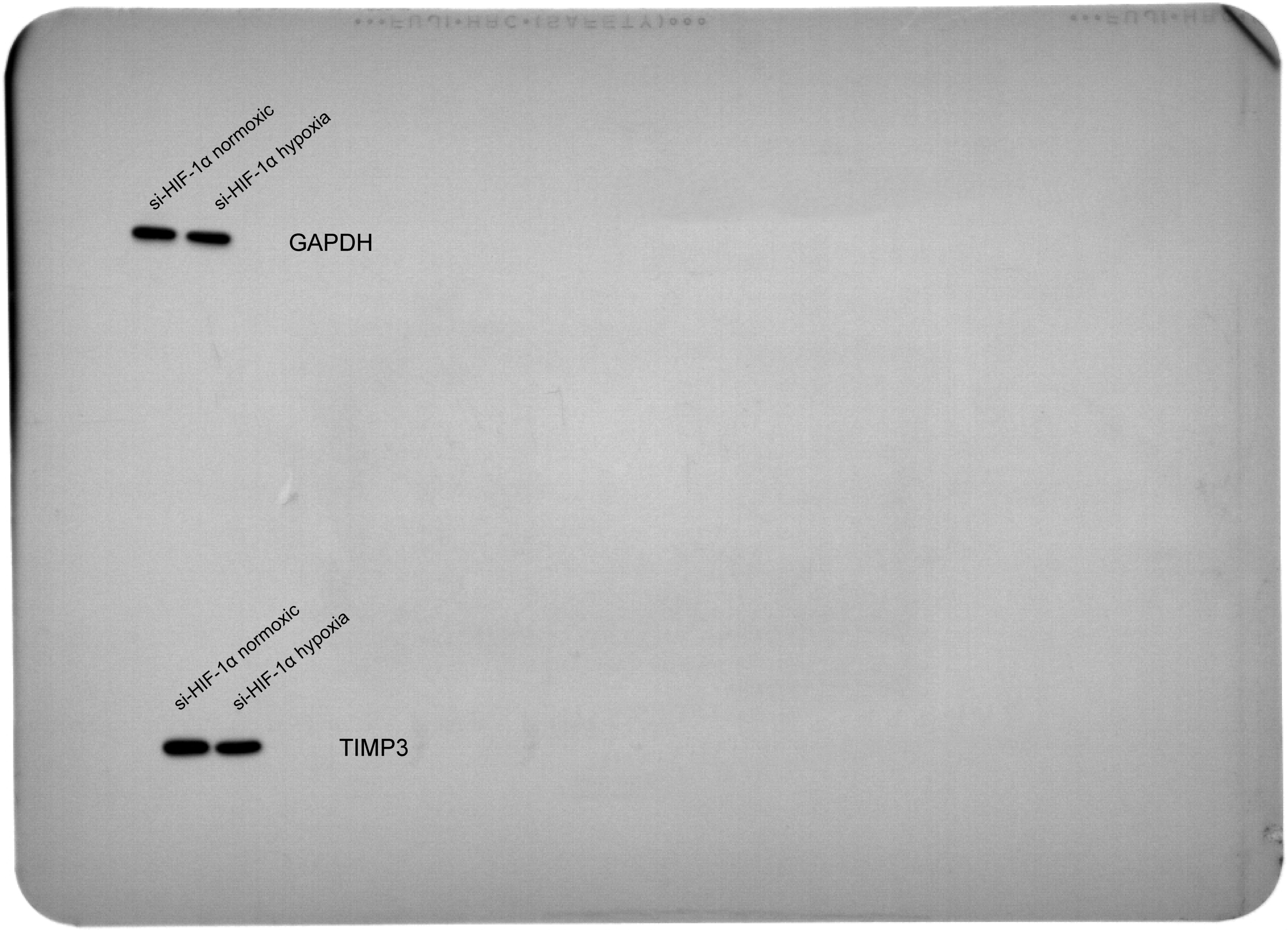
si-HIF-1α normoxic
si-HIF-1α hypoxia
GAPDH
si-HIF-1α normoxic
si-HIF-1α hypoxia
TIMP3
